# Helicase Lymphoid-Specific Enzyme Contributes to the Maintenance of Methylation of SST1 Pericentromeric Repeats That Are Frequently Demethylated in Colon Cancer and Associate with Genomic Damage

**DOI:** 10.3390/epigenomes1010002

**Published:** 2016-09-22

**Authors:** Johanna K. Samuelsson, Gabrijela Dumbovic, Cristian Polo, Cristina Moreta, Andreu Alibés, Tatiana Ruiz-Larroya, Pepita Giménez-Bonafé, Sergio Alonso, Sonia-V. Forcales, Perucho Manuel

**Affiliations:** 1 Sanford-Burnham-Prebys Medical Discovery Institute, La Jolla, CA 92037, USA; 2 Active Motif, 1914 Palomar Oaks Way, Suite 150, Carlsbad, CA 92008, USA; 3 Institute of Predictive and Personalized Medicine of Cancer (IMPPC), Institut d’Investigació en Ciències de la Salut Germans Trias i Pujol (IGTP), Campus Can Ruti, Badalona 08916, Barcelona, Spain; 4 Departament de Ciències Fisiòlogiques, Facultat de Medicina i Ciències de la Salut, Campus Ciències de la Salut, Bellvitge, Universitat de Barcelona, Hospitalet del Llobregat 08916, Barcelona, Spain; 5 Institució Catalana de Recerca i Estudis Avançats (ICREA), Barcelona 08010, Spain

**Keywords:** DNA demethylation, epigenetics, satellite element, colorectal cancer

## Abstract

DNA hypomethylation at repetitive elements accounts for the genome-wide DNA hypomethylation common in cancer, including colorectal cancer (CRC). We identified a pericentromeric repeat element called SST1 frequently hypomethylated (>5% demethylation compared with matched normal tissue) in several cancers, including 28 of 128 (22%) CRCs. SST1 somatic demethylation associated with genome damage, especially in tumors with wild-type *TP53.* Seven percent of the 128 CRCs exhibited a higher (“severe”) level of demethylation (≥10%) that co-occurred with *TP53* mutations. SST1 demethylation correlated with distinct histone marks in CRC cell lines and primary tumors: demethylated SST1 associated with high levels of the repressive histone 3 lysine 27 trimethylation (H3K27me3) mark and lower levels of histone 3 lysine 9 trimethylation (H3K9me3). Furthermore, induced demethylation of SST1 by 5-aza-dC led to increased H3K27me3 and reduced H3K9me3. Thus, in some CRCs, SST1 demethylation reflects an epigenetic reprogramming associated with changes in chromatin structure that may affect chromosomal integrity. The chromatin remodeler factor, the helicase lymphoid-specific (HELLS) enzyme, called the “epigenetic guardian of repetitive elements”, interacted with SST1 as shown by chromatin immunoprecipitation, and down-regulation of *HELLS* by shRNA resulted in demethylation of SST1 in vitro. Altogether these results suggest that HELLS contributes to SST1 methylation maintenance. Alterations in HELLS recruitment and function could contribute to the somatic demethylation of SST1 repeat elements undergone before and/or during CRC pathogenesis.

## 1. Introduction

Somatic epigenetic changes are common hallmarks of cancer. Epigenetic alterations include global DNA hypomethylation, hypermethylation of CpG islands, chromatin alterations, and loss of imprinting [1–3]. Global genomic hypomethylation has been found in virtually every tumor type studied, both benign and malignant [3]. It also has been linked to oncogene-specific activation, disruption of genomic imprinting, chromosomal alterations, and increased tumor frequency in mouse models in vitro and in vivo [4–7].

Global DNA hypomethylation observed in cancer primarily reflects somatic demethylation of DNA repetitive elements, which account for a large fraction (around 40%) of the human genome [8]. Hypomethylation has been reported to be particularly severe in pericentromeric heterochromatin [9–11], and is known to result in centromere decondensation, enhancing chromosome recombination [12,13]. In addition, in normal cells, DNA methylation stabilizes chromosomes by silencing non-coding DNA and transposable DNA elements [14]. All of these data indicate that DNA hypomethylation of these repeat sequences might alter proper mitotic processes, and lead to deletions, translocations, and chromosomal rearrangements [2,5].

Chromosomal abnormalities associated with DNA hypomethylation have also been observed in other diseases distinct from cancer. For instance, in all tissues analyzed from ICF syndrome (immunodeficiency, centromeric region instability, facial abnormalities), hypomethylation of satellite sequences found in a subset of chromosomes is accompanied by instability at juxtacentromeric heterochromatin in the same chromosomes [8,12,15], further substantiating the causative link between hypomethylation and chromosome alterations.

Both DNA hypo- and hypermethylation alterations have also been associated with aging, a well-established risk factor for cancer. Accordingly, a progressive loss of overall methylation has been found during in vitro culture of fibroblasts [16], in aging animals and humans [17–19]. Different types of interspersed repetitive sequences appear to be targeted to varying degrees of age-associated hypomethylation [20], indicating that both age-dependent and independent mechanisms can contribute to global hypomethylation in cancer.

We previously found that global hypomethylation increases with patient age and correlates with genomic damage in colorectal cancer (CRC) [21]. Based on these findings, we proposed a “wear and tear” model linking aging, epigenetic somatic errors, and cancer. The model is based on the consideration that during the task of replicating the three billion base pairs of the human genome, errors in replication of DNA and its methylation patterns unavoidably accumulate because evolution by natural selection did not provide for DNA methylation repair mechanisms, as it did for mutations [22]. DNA demethylation can facilitate mitotic errors when reaching a significant level, especially in pericentromeric regions [6,23–25].

During our previous studies we observed a particular sequence that exhibited a prominent demethylation in around 20% of gastrointestinal tumors [21]. This sequence corresponds to a pericentromeric tandem repeat that belongs to the SST1 family according to RepeatMasker [26]; it preferentially maps to acrocentric chromosomes and has also been called NBL2 [27,28]. Here, we have investigated if SST1 sequences could serve as markers for demethylation. We also studied whether SST1 demethylation was linked to changes in histone post-translational modifications and alterations in chromatin factors that could be involved in its demethylation.

## 2. Results

### 2.1. Demethylation of SST1 Pericentromeric Satellite Repeats in Human Cancers

**Figure 1.**
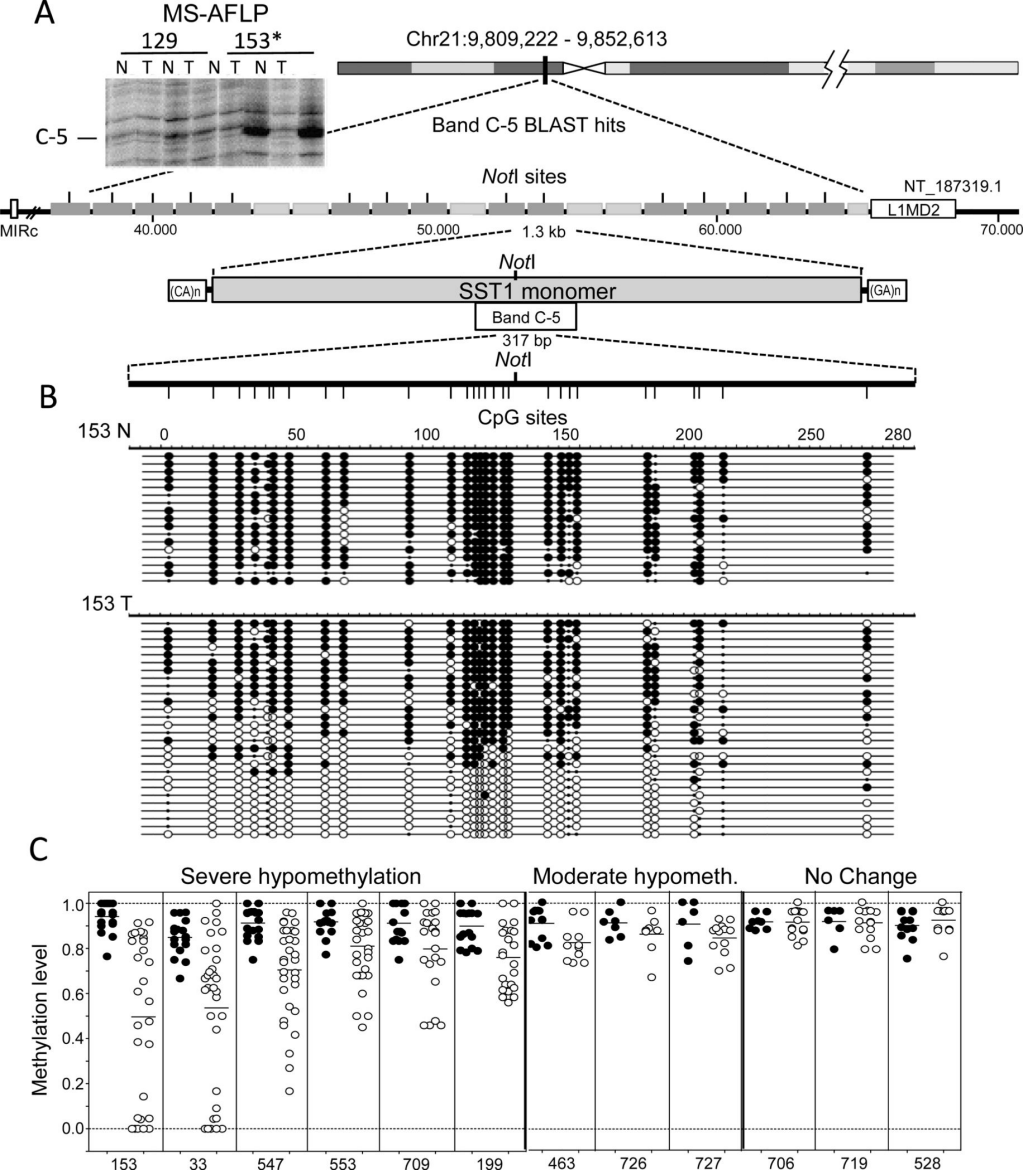
SST1 elements are demethylated in colorectal cancer. (**A**) DNA fingerprinting by methylation sensitive-amplified fragment length polymorphism (MS-AFLP) identified a hypomethylated region in 22% of colorectal cancer (CRC) tumors (band C-5). The insert depicts the region of the fingerprint of two CRC cases, showing the intense bandC-5 in tumor 153, indicating demethylation of the NotI site. Band C-5 correspond (bed to a pericentromeric region on chromosome 21, which contains 21 SST1 satellite tandem repeats. Most of these SST1 elements contain a NotI site; that can be detected by MS-AFLP. Each repeat of approximately 1.3 kb (grey rectangles) is flanked by CA- and GA-rich regions. On each side of the21 repeat region, families of retrotransposons are present (MIRC and LIM2). N and T: normal and tumor tissue DNA; (**B**) A region of 317 bp comprising 28 CpG sites and the NotI site was analyzed by bisulfite sequencing in several CRCs. Analysis of case 153 is represented, where black and white circles represent methylated and unmethylated CpG sites, respectively. Small black dots indicate polymorphisms including TpA sequences possibly resulting from spontaneous deamination of methylated CpGs on the opposite DNA strand. Each row represents the sequence of an individual plasmid clone; and (**C**) methylation status of SST1 satellites from chromosome 21 in 12 CRCs cases. SST1 methylation levels indicate the methylation average of the 28 CpGs analyzed by bisulfite sequencing from individual PCR clones (rows in (**B**)) in colon normal samples (black) and tumor samples (white). Severe demethylated cases show a methylation average difference between normal and tumor equal or higher than 10%, whereas moderate demethylation cases show a difference between 5% and 10%.

A previous genome-wide analysis of DNA methylation levels by a DNA fingerprint technique, methylation sensitive-amplified fragment length polymorphism (MS-AFLP), identified a frequently hypomethylated sequence (band C-5 in Figure 1, upper left) in colorectal (CRC) and gastric tumors [21]. Since the locus exhibited the stronger demethylation signal in the fingerprints, we decided to characterize and study in detail its features. A BLAST search returned several hits on chromosome 21, each one embedded in one of 21 tandem repeats, belonging to a SST1 element, a moderately repeated pericentromeric satellite sequence according to the RepeatMasker database [26] (Figure 1A). Tumors found demethylated by MS-AFLP displayed clear levels of demethylation as estimated by bisulfite sequencing(Figure 1B).

SST1 hypomethylation was observed in 22% of CRCs, compared to their corresponding normal tissue, from a panel of 128 randomly selected CRCs. SST1 methylation levels were also analyzed in CRC and ovarian cancer cell lines, as well as in breast, ovary and gastric primary cancers. Table S1 shows that SST1 demethylation also occurred in all of these cancers.

The average level of global DNA demethylation in human CRC ranges between eight and ten percent [29], in contrast with the high range variation of demethylation observed in SST1 elements (Figure 1C). We then classified SST1 demethylated tumors as displaying “moderate” or “severe” demethylation if they exhibited an average methylation level of ≥5% <10% (moderate), or ≥10% (severe), below that observed in their corresponding normal tissues (Figure 1C). Tumors with demethylation levels lower than 5% were considered negative for somatic demethylation. The distribution of somatic SST1 methylation changes along the informative cases analyzed by bisulfite sequencing is shown in Figure S1. The results show the vast majority of cases following a normal distribution, with a few cases in the high end of demethylation deviating from normality. They correspond to the severe demethylation group.

### 2.2. SST1 Demethylation as a Marker of Global Hypomethylation

Demethylation of long interspersed nuclear element-1 (LINE-1) accounts for most of the global DNA hypomethylation observed in cancer [8] and is a commonly-used marker of global hypomethylation [30,31]. The methylation status of LINE-1 was analyzed by bisulfite sequencing in a subset of CRCs previously analyzed for SST1 methylation. LINE-1 demethylation correlated with SST1 demethylation (*r*^2^ = 0.473, *p* = 0.0008) (Figure S2A), showing that SST1 and LINE-1 demethylation overlap.

To determine whether demethylation of SST1 sequences was restricted to chromosome 21 we designed specific primers unique to chromosomes 7 and 9, and analyzed methylation levels by bisulfite sequencing. SST1 also displayed demethylation at these chromosomes (Figure S2B). These results show that SST1 demethylation is not a local event specific to chromosome 21, but affects other chromosomes as well.

### 2.3. Association of SST1 Demethylation with Genomic Damage

**Figure 2.**
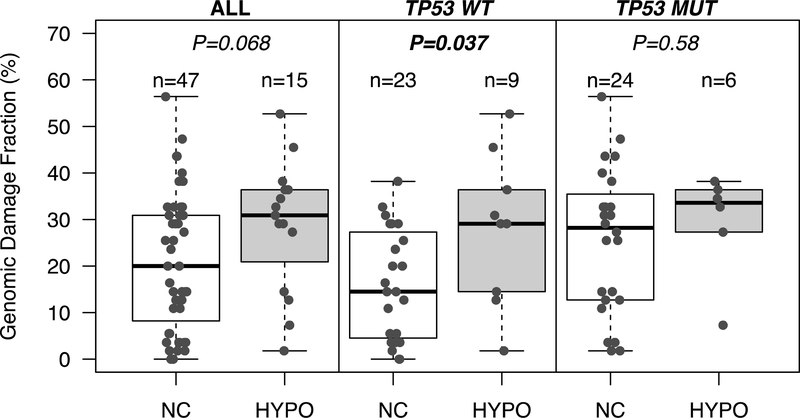
Demethylation of SST1s is associated with genomic damage in CRCs with wild-type (WT) *TP53*, but not with mutant (MUT) *TP53*. *p* values were calculated by *t* test. Genomic damage fraction (GDF) was estimated by AP-PCR DNA fingerprinting as previously described [21]. GDF indicates average number of copy number changes (losses and gains) in tumor relative to normal tissue. NC: no change in DNA methylation; HYPO: DNA hypomethylation.

In gastrointestinal cancers, DNA hypomethylation associates with DNA copy number alterations [21]. Therefore, we further investigated whether SST1 demethylation correlated with increased levels of genomic damage, expressed as genomic damage fraction (GDF). GDF had been estimated in many of these CRC samples in a previous study in which DNA copy number changes were analyzed by arbitrarily primed PCR (AP-PCR) [21], a DNA fingerprinting technique that estimates global genomic alterations [32,33]. While *TP53* wild-type (WT) tumors with SST1 hypomethylation had similar level of GDF than *TP53* mutant (MUT) tumors (with or without hypomethylation), within *TP53* WT tumors there was a significantly higher GDF in SST1 hypomethylated tumors (Figure 2).

### 2.4. SST1 Demethylation and Genotype/Phenotype of CRC

**Figure 3.**
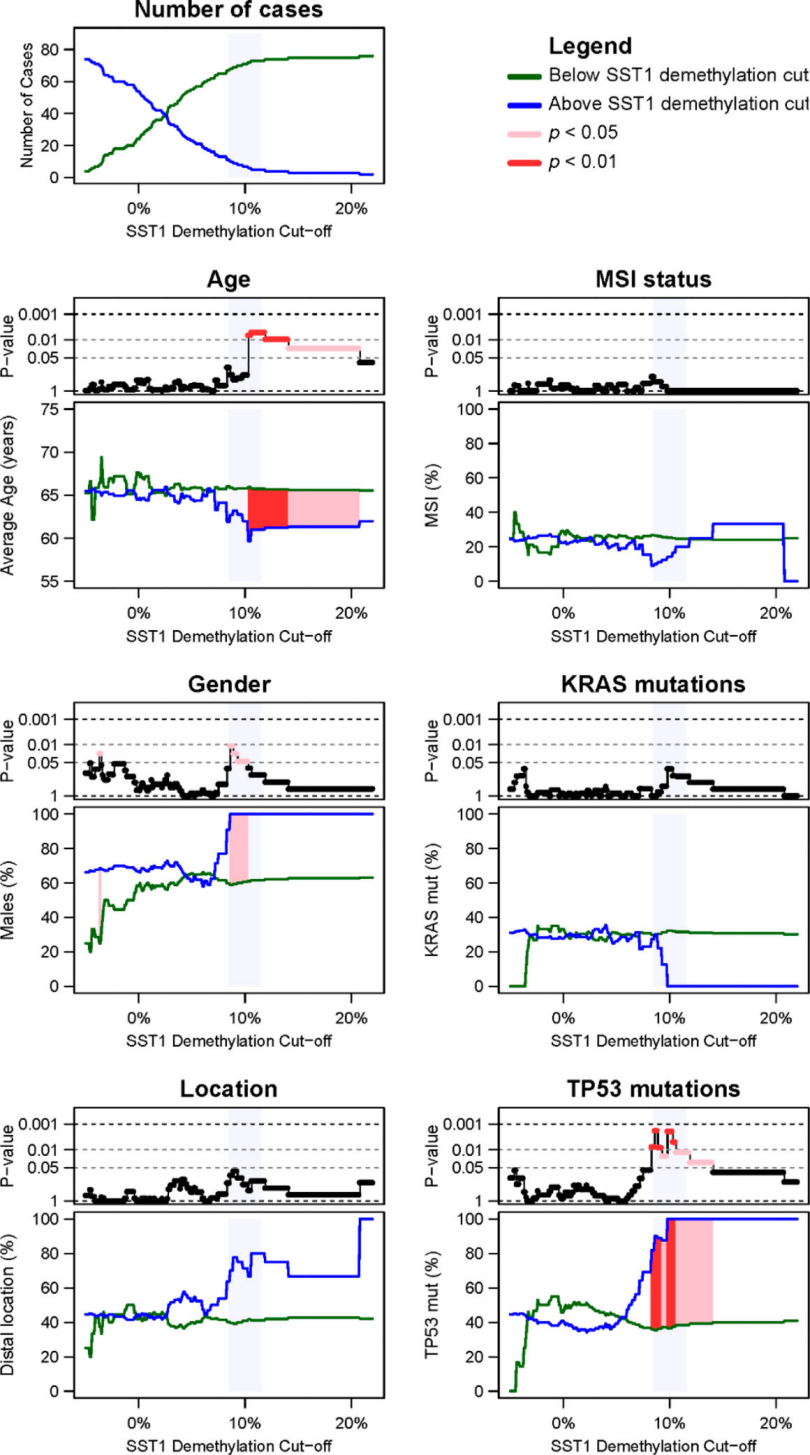
SST1 methylation and clinicopathological features of CRC. CRC cases with quantitative information of SST1 somatic demethylation (*n* = 80) were classified into two groups, i.e., below (green lines) and above (blue lines) increasing SST1 demethylation cutoff values. We explored the effect of the different cutoffs between −5% and 22%, with a 0.1% increment. The information of every clinicopathological and mutational factor is represented in a stacked pair of graphs. The upper graph of every pair shows, in a negative logarithmic scale (*y* axis), the *p* value of the comparison between tumors below and tumors above the SST1 demethylation cutoff employed for the classification (*x* axis). In pink, *p* values < 0.05. In red, *p* values < 0.01. The lower graph of every pair shows the values of the parameters (*y* axis) for the group of tumors below (green) or above the cutoff (blue). The SST1 demethylation regions where the applied cutoff yielded statistical significance are also indicated in pink (*p* < 0.05) and red (*p* < 0.01). Age was compared by Student’s *t* test. Gender, *KRAS* mutations and *TP53* mutations were analyzed by Fisher’s exact test. Shaded in light blue, the region around the 10% demethylation cutoff.

To evaluate the impact of SST1 hypomethylation in CRC genotype and phenotype, we analyzed its association with clinicopathological and molecular parameters in the same panel of 128 CRCs. We compared three groups of tumors as negative for demethylation (<5%), moderate (≥5% <10%), and severe (≥10%) SST1 demethylation (Table S2). The fluctuation in *p* values in the comparison between clinicopathological and molecular variables in CRC according to the SST1 somatic demethylation cutoff employed for the classification is shown in Figure 3.

No significant differences were found between tumors with and without overall SST1 hypomethylation (NC vs. rest) and any of the clinicopathological and molecular parameters studied (Table S2). However, comparison of severe demethylation with other groups showed several asymmetries. Moderate SST1 demethylation appeared more frequent in older (≥65) females, while severe demethylation in younger (≤64) males. Moderate demethylation tended to be more frequent in proximal tumors while severe demethylation in distal tumors (Figure 3, Table S2). Regarding genotype, severe SST1 demethylation was significantly more common in patients with mutated *TP53*, (Figure 3, Table S2). The significance remained across several cutoff values around the 10% zone that is coincident with the change in slope of the methylation distribution reflecting a deviation of normality (Figure S1). In tumors with severe SST1 hypomethylation, *TP53* and *KRAS* mutations seemed to be mutually exclusive (8 MUT and 1 WT for *TP53* vs. 1 MUT and 8 WT for *KRAS*, *p* = 0.003).

### 2.5. SST1 Hypomethylation Associates with Histone H3 Lysine 27 Trimethylation in Vitro and in Vivo

**Figure 4.**
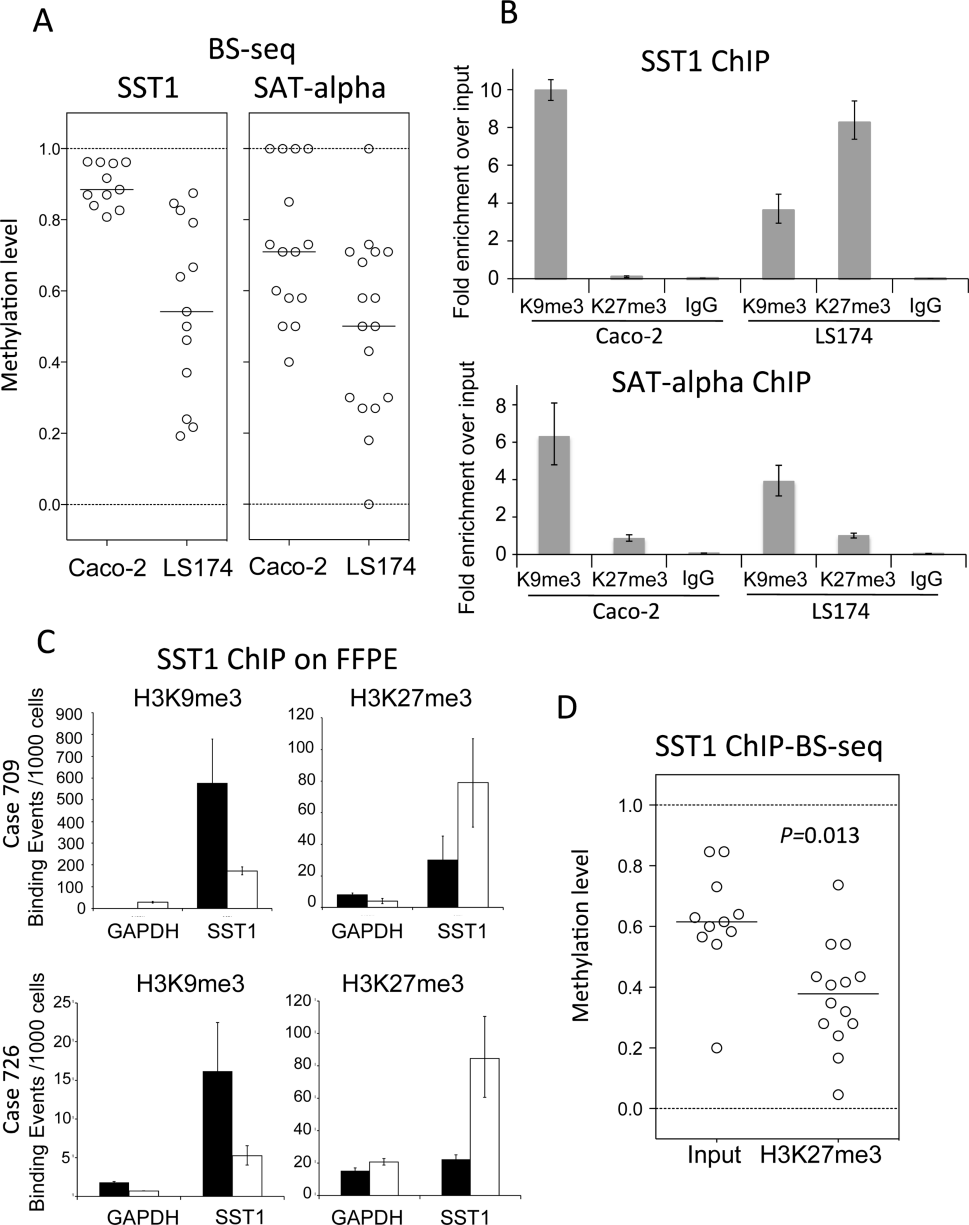
SST1, but not SATα methylation, correlates positively with histone 3 lysine 9 trimethylation (H3K9me3) levels and negatively with histone 3 lysine 27 trimethylation (H3K27me3). (**A**) SST1 and SATα methylation levels of individual clones analyzed by bisufite sequencing in CIRC cell lines Caco-2 and LS174. As in Figure 1C, each dot corresponds to average methylation level present in one PCR clone sequence; (**B**) chromatin immunoprecipitation analysis (ChIP) of histone modifications on SST1 element. Demethylation of SST1 associates with increased H3K27me3 and reduction of H3K9me3 in SST1 chromatin. Demethylation of SATα element associates with decrease of H3K9me3 but not with H3K27me3 increase. Fold change was calculated relative to the input (comparative Ct method). IgG indicate immunoprecipitation background levels; (**C**) ChIP analysis of histone modifications associated with SST1 repetitive elements in two representative cases of colon cancer formalin-fixed paraffin-embedded (FFPE) primary tissue. Both tumors (709 and 726) displaying severe and moderate SST1 demethylation, respectively (Figure 1C), showed a shift from high levels of SST1 methylation and H3K9me3 enrichment in normal tissue (black bars) to SST1 demethylation accompanied with H3K9me3 reduction and H3K27me3 increase in the tumor (white bars); and (**D**) demethylated SST1 DNA associates with H3K27me3 by ChIP analysis in CRC cell line LS174T. The immunoprecipitated genomic DNA regions were bisulfite sequenced to monitor SST1 (ChIP-BS-seq). Input DNA: bisulfite treated input DNA purified after sonication. H3K27me3 DNA: bisulfite-treated DNA after H3K27me3 immunoprecipitation. SST1 elements associated with H3K27me3 show significantly higher demethylation compared to the input. *p* value was calculated by *t* test.

Hypomethylation of heterochromatin at pericentromeric satellite sequences results in centromeric decondensation [12,13]. Histone post-translational modifications also play a crucial role in chromatin regulation and organization [34]. The association between SST1 demethylation and increased levels of genomic damage suggested that demethylation of SST1 satellites could affect chromatin architecture. Therefore, we explored whether changes in SST1 methylation associated differentially with specific histone marks. Colorectal and ovarian cancer cell lines with variable levels of SST1 methylation (Figure 4A, Figure S3A) were analyzed by chromatin immunoprecipitation (ChIP) to monitor the presence of several histone H3 marks in the SST1 region (Figure 4B, Figure S3B).

Colon and ovarian cancer cell lines characterized by highly methylated SST1 elements (Caco-2 and A2780ADR, Figure 4A, and Figure S3A) showed high levels of histone 3 lysine 9 trimethylation (H3K9me3) mark and low levels of histone 3 lysine 27 trimethylation (H3K27me3) in SST1 repeats (Figure 4B, Figure S3B). The same behavior was found in another satellite repeat, SATα (Figure 4A,B). In contrast, cell lines with SST1 demethylation (LS174T and OV90), exhibited enrichment of H3K27me3, but lower levels of H3K9me3. This epigenetic profile was specific for SST1 elements since SATα did not show increase in H3K27me3 (Figure 4B).

To test if the epigenetic profile observed in vitro was also found in vivo, we performed ChIP in formalin-fixed paraffin-embedded (FFPE) samples. Two informative cases are shown in Figure 4C. FFPE-ChIP validated the observed shift from high levels of DNA methylation and H3K9me3 at SST1 in normal tissues to a reduction of H3K9me3 and enrichment of H3K27me3 in tumors exhibiting SST1 demethylation (cases 709 and 726, see Figure 1). Thus, a highly stable repressive state (high levels of DNA methylation and H3K9me3) is replaced by a more plastic state (low levels of DNA methylation and high levels of H3K27me3) also in primary CRC.

To further analyze the association between SST1 hypomethylation and H3K27me3 enrichment, as well as to clarify whether the two events truly coincide at the same loci, chromatin from LS174T was immunoprecipitated with anti-H3K27me3 and the eluted DNA was bisulfite sequenced. SST1 sequences interacting with H3K27me3 showed reduced levels of methylation compared to their inputs (Figure 4D).

### 2.6. Induced SST1 Demethylation Leads to Increased H3K27me3 Levels

**Figure 5.**
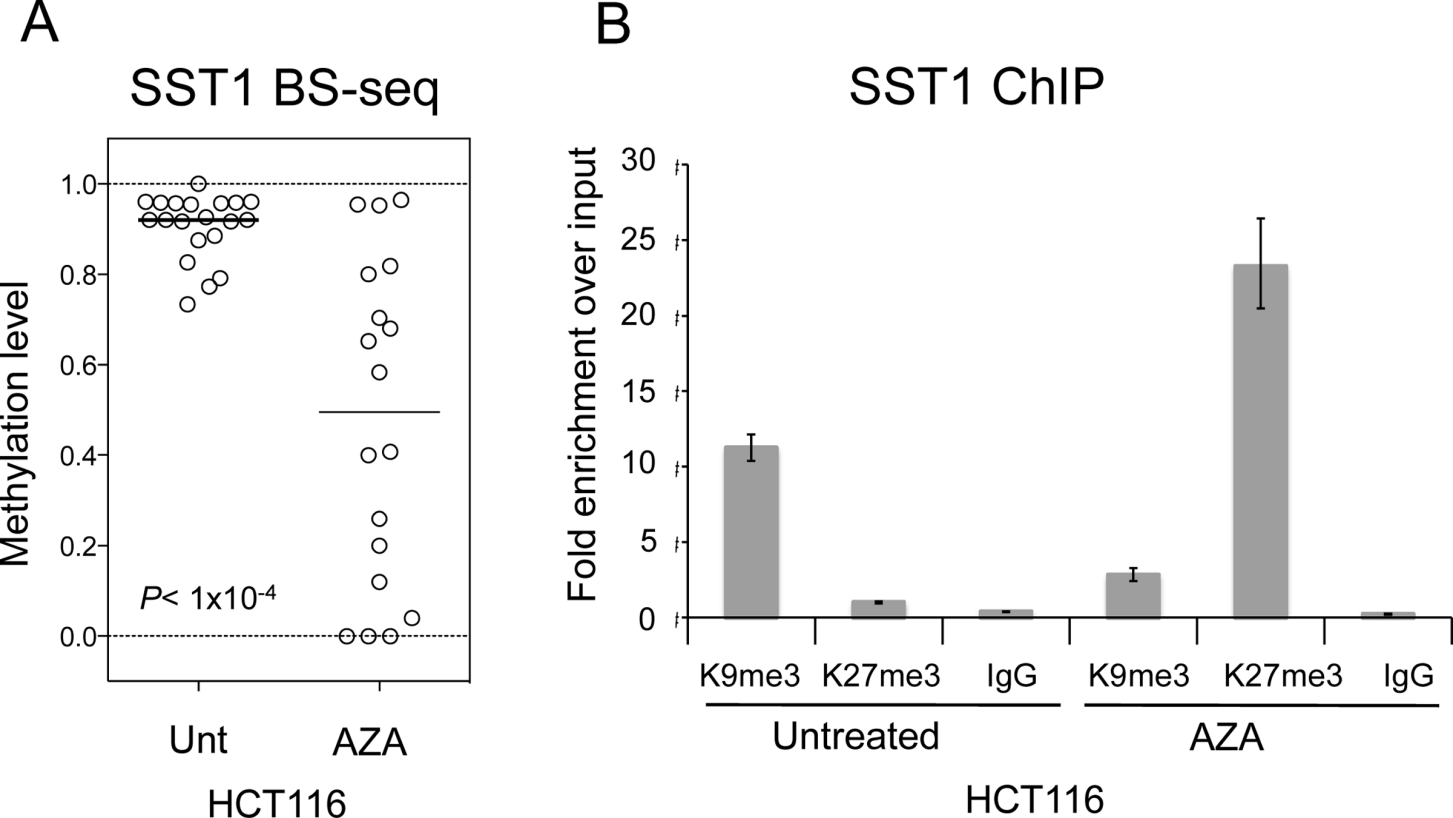
SST1 demethylation changes histone modifications in CRC] cell line HCT116. (**A**) SST1 methylation levels of individual clones estimated by bisulfite sequencing. Untreated (Unt) or treated with 5-aza-dC at 2.5 μM (AZA); and (**B**) SST1 demethylation, induced by 5-aza-dC (AZA) treatment (2.5 mM), is followed by an increase in H3K27me3 levels as shown by chromatin immunoprecipitation. H3K9me3 and H3K27me3 levels shown as fold enrichment relative to the input. IgG indicate immunoprecipitation background levels.

We have shown a co-occurrence of increased levels of H3K27me3 with reduced levels of SST1 DNA methylation. To investigate whether these epigenetic events are mechanistically linked and to get insight into the order of events, we treated a highly methylated CRC cell line, HCT116, with a specific DNA methyltransferase inhibitor 5-aza-2-deoxycitidine (5-aza-dC) to induce global DNA demethylation. Bisulfite sequencing of 5-aza-dC-treated cells confirmed an induced heterogeneous hypomethylation of SST1 (Figure 5A). A subsequent ChIP analysis of 5-aza-dC-treated HCT116 cells revealed that coincident with reduced levels of DNA methylation there was a decrease in H3K9me3 and an increase in H3K27me3 (Figure 5B).

### 2.7. A Chromatin Remodeling Factor, HELLS, Maintains the Methylated SST1 Region

**Figure 6.**
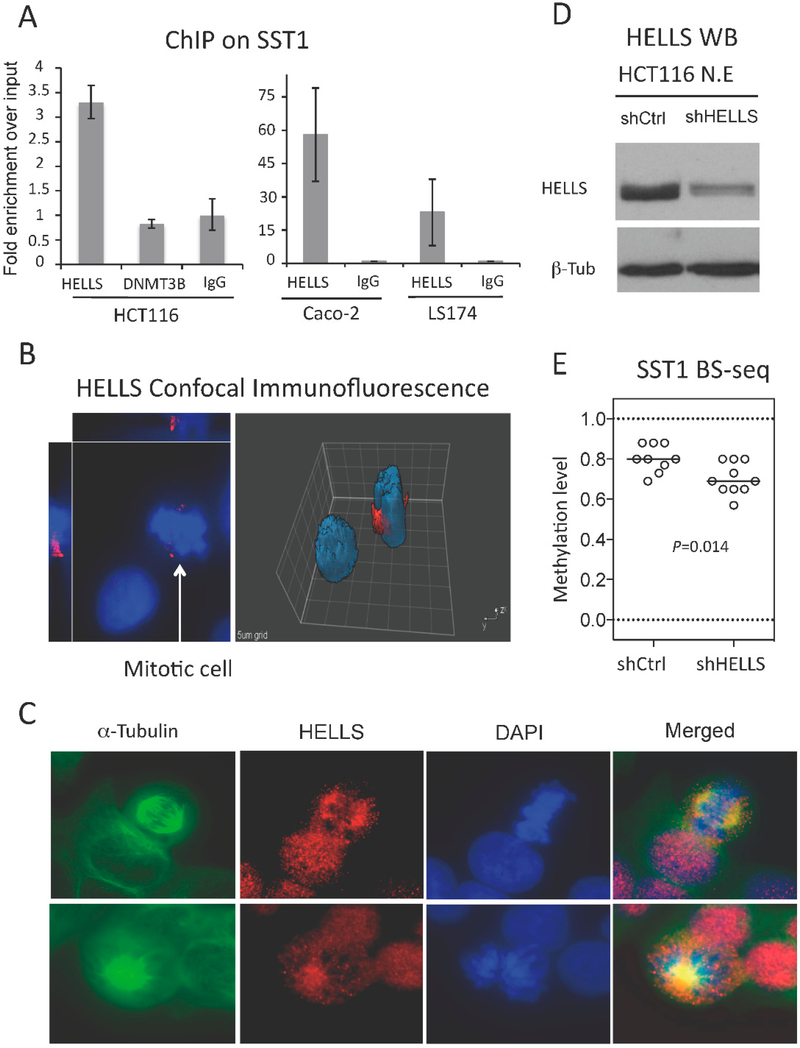
HELLS is recruited to SST1 regions, localizes in the centrosome and the mitotic spindle, and HELLS knockdown results in SST1 demethylation. (**A**) ChIP analysis of HELLS and DNA methyltransferase 3B (DNMT3B) recruitment to SST1 repetitive elements in CRC cell line HCT116 (left graph). The right graph shows HELLS recruitment to SST1 monitored by ChIP in Caco-2 (SST1 methylated) and LS174 (SST1 demethylated). Relative recruitment was calculated by the comparative Ct method and the input values were used as normalizers. IgG shows art immunoprecipiation background; (**B**) HELLS expression by confocal immunofluorescence. Caco-2 cells stained with primary antibody against HELLS (red) and counterstained with 4′,6-diamidino-2-phanylindole (DAPI) (blue). Image analysis by Slidebook software v4 (Intelligent Imaging Innovations, Denver, CO, USA) shows increased protein levels of HELLS around what seems to be the centrosome regions of mitotic cells; (**C**) Co-immunofluorescence of HELLS with α-tubulin, a marker of the mitotic spindle, in HCT116 cells; (**D**) Western blot on nuclear extracts from HCT116 polyclones obtained after lentiviral infection of small hairpin RNAs (shRNA) of a scrambled control sequence (shCtrl) or targeting HELLS (shHELLS); and (**E**) SST1 methylation levels of individual clones analyzed by bisulfite sequencing in colon cancer cell line HCT116 with scrambled shRNA (shCtrl) or shRNAs for HELLS (shHELLS). This analysis was performed three weeks post-infection. *p* value was calculated by Mann Whitney test.

As mentioned earlier, the severe SST1 demethylation was observed in relatively younger patients, thus not fitting our “wear and tear” model. To gain insight into an alternative mechanism contributing to SST1 demethylation, we studied by ChIP the recruitment of two factors known to influence DNA methylation at repetitive elements, such as HELLS (also known as PASG for proliferation associated SNF2-like gene) and the DNA methyltransferase 3B (DNMT3B).HELLS is a SWI/SNF helicase related to the SNF2 family of chromatin-remodeling ATPases that has been shown to play a fundamental role in glob ai DNA methylation, especially at pericentromeric regions and repetitive elements [35–38], for which it is called the “epigenetic guardian of repetitive elements” [39]. ChIP in HCT116, which contains a highly methylated SST1, shows recruitment of HELLS, but not of DNMT3B (Figure 6A left). Furthermore, HELLS recruitment was different in two CRC cell lines exhibiting variable levels of SST1 methylation; HELLS was recruited more to highly-methylated SST1 elements (Caco-2) than to de methylated SST1 elements (LS174T) (Figure 6A right). Immunofluorescence analysis showed a nuclear localization of HELLS with no specific distribution pattern except: in mitotic nuclei where it seemed to show a preferential localization to centrosomic regions (Figure 6B) Immunostaining with anti-α-tubulin antibody showed co-localization of HELLS and the mitofic spindle (Figure 6C).

*HELLS* expression by RT-PCR correlated negatively with SST1 demethylation (Figure S4). *HELLS* expression levels were relatively low in primary tumors exhibiting severe SST1 demethylation such as CRCs 33, 55, 547, and 553 (Figure S4A). Moreover, colorectal and ovarian cancer cell lines exhibiting low levels of PST1 methylation (LS174T and OV90 in Figure 4A and Figure S3), also displayed low *HELLS* RNA levels (Figure S4B). This correlation was significant for the CRC cell lines analyzed (Figure S4C,D).

To validate HELLS involvement in maintaining SST1 methylation, we used a small hairpin RNA (shRNA)-mediated approach to stably downregulate *HELLS* expression and determined the subsequent effect on SST1 methylation levels. Two CRC cell lines (Caco-2 and HCT116, with high levels of *HELLS* expression and SST1 methylation) were infected with shRNA-containing lentiviral vectors and puromycin-resistant polyclones were selected from a control scrambled shRNA sequence (shControl) and from a shRNA against HELLS (shHELLS). *HELLS* downregulation was verified by RT-qPCR (not shown) and by Western blots in nuclear extracts (Figure 6D). After downregulation of *HELLS* expression was confirmed, SST1 methylation levels were monitored by bisulfite sequencing in HCT116 shControl and shHELLS cells. Cells with reduced levels of *HELLS* exhibited significantly lower levels of SST1 methylation (70.3% average SST1 methylation compared to control 80.3% average SST1 méthylation, *p* = 0.014) (Figure 6E). These results show that HELLS contributes to the maintenance of SST1 methylation.

## 3. Discussion

We have shown that demethylation of SST1 elements occurs in CRC, gastric, ovarian, and breast cancers. Other groups have also shown that demethylation of this same repetitive sequence, which is also called NBL2 [27], is present in neuroblastomas [26], hepatocellular carcinomas [40], and gastric tumors [41]. We also found that tumors with SST1 hypomethylation seemed to present higher genomic damage than tumors without. Although borderline, this tendency became more evident if only tumors without *TP53* mutations were considered (Figure 2).

The negative association between demethylation and *TP53* mutations was reversed in a minority (≈7%) of tumors showing a more “severe” demethylation (Table S2). Despite the small number of cases, the association of “severe” SST1 demethylated tumors with *TP53* mutations held after applying different cutoffs for the distinction of severe vs. moderate demethylation (Figure 3). Thus, our data suggests that DNA demethylation may play a role in facilitating genomic instability through two different mechanisms. One may involve “moderate” SST1 demethylation without association with *TP53* mutations that fits the “wear and tear” model [21]. Another mechanism may occur in a minority of tumors with *TP53* mutations that accumulate “severe” SST1 DNA hypomethylation in an age-independent manner.

We also show the specific epigenetic reprogramming of SST1 repetitive elements, where DNA demethylation is accompanied by accumulation of H3K27me3, a polycomb complex 2 (PRC2)-catalyzed modification. Antagonism between DNA methylation and H3K27me3, two repressive marks, can appear contradictory at first. While other groups have also shown a mutually exclusive relationship between H3K27me3 and DNA methylation [42–47], several reports support a functional interdependence between DNA methylation and H3K27me3. For instance, polycomb directs de novo DNA methylation in cancer cells with enhancer of zeste homolog 2 (EZH2) serving as a recruitment platform for DNA methyltransferases as a possible mechanism [43,47,48]. In some cancers, an aberrant methylation of genes originally silenced by the PRC mark H3K27me3 has been called an epigenetic switching [43]. Instead, we observed an opposite event, a sort of “reverse switching”, an epigenetic reprogramming whereby DNA methylation is replaced by H3K27me3 upon its demethylation.

Our findings are in agreement with the observation that DNA methylation is incompatible with PRC2 occupancy [45,49] and that H3K27me3-associated CG-dense regions are highly demethylated in CRC cells and in embryonic stem (ES) cells [50] Furthermore, our results not only show an antagonistic correlation between H3K27me3 and DNA methylation at SST1 elements, but SST1 demethylation induced by 5-aza-dC treatment also resulted in H3K27me3 increase at SST1 loci. This temporal mechanistic interrelationship has also been reported in *DNMT* triple knockout ES cells which show global loss of CpG methylation associated with the formation of broad local enrichments for H3K27me3 [50]. This suggests that DNA methylation restricts H3K27me3 deposition and that, somehow, absence of DNA methylation allows the polycomb mark to spread. This is also in accordance with other reports showing that drug-induced demethylation at selected loci was accompanied by a partial loss of chromatin domain boundaries and the spreading of inactive H3K27me3 mark [51].

Since SST1 is a pericentromeric repetitive element and the maintenance of heavy methylation in these sequences has been shown to be critical for correct chromosomal replication [52,53], to maintain SST1 repressed at all times may be advantageous for cell viability. One can hypothesize that following the loss of DNA methylation in these pericentromeric repeats before and/or during the tumorigenic process, the epigenetic machinery deposits, by a yet unclear mechanism, the H3K27me3 polycomb repressive mark as a means to maintain the silenced region.

SST1 is a repetitive element, part of constitutive heterochromatin. In this regard, a mechanistic tie between H3K9me3 and DNA methylation has been observed at constitutive heterochromatin regions. For instance, centromeres and telomeres show high levels of DNA methylation and H3K9me3 [54,55]. This association between DNA methylation and H3K9me3 is also present in pericentric satellite repeats in mouse ES cells where DNMT3B-dependent DNA methylation is directed by H3K9me3 [56].

Altogether, our data suggests that SST1 elements lose heterochromatin features (DNA methylation and H3K9me3) and are reprogrammed to a facultative chromatin acquiring a more relaxed architecture (gain of H3K27me3). The mechanism underlying these epigenetic changes during CRC development is unclear, but presumably entails changes in expression, chromatin recruitment, or activity of enzymes involved directly or indirectly in DNA methylation. Supporting this hypothesis, we describe how HELLS, a chromatin-associated factor, appears to contribute to SST1 DNA methylation maintenance.

Although a *HELLS* splicing variant has been described in non-small cell lung cancer [57], The Cancer Genome Atlas (TCGA) [58] data shows a very low frequency for *HELLS* mutations in CRC. However, SST1 methylation correlates with *HELLS* expression in CRC tumors and cell lines. Functional assays also show that *HELLS* downregulation decreases SST1 methylation. Altogether these results support the idea that if an aberrantly low level of recruitment happens in vivo before and/or during tumorigenesis, it could contribute to SST1 demethylation.

Other groups have recently shown that HELLS is required to maintain methylated long terminal repeats (LTRs) and several families of satellite repeats and LINEs during mouse embryonic development [59]. HELLS-mediated methylation of satellites and retrotransposons is DNMT3B-independent and, along with other experimental data, supports a de novo methylation role for HELLS in a specific time frame during embryogenesis. Whether in human cells HELLS follows a similar mechanism is not known, but the absence of DNMT3B at SST1 repeats suggests it could be similar.

Very little is still known about the genomics and epigenomics of repetitive elements and how these could have an impact on diverse functions and ultimately on cell behavior [60]. Difficulty in sequencing and, therefore, analyzing repetitive regions, along with the classical idea that repetitive DNA is “junk”, has discouraged research on this field. However, new technologies, such as single molecule real-time sequencing (SMRT), that allow obtaining longer reads, may help to overcome these limitations and to fill the gap in our knowledge on repetitive sequences and their roles [61,62].

In summary, our data suggest that the epigenetic reprogramming at SST1 regions may contribute to genomic instability by facilitating aberrant mitosis and chromosome segregation defects. Because both DNA hypermethylation and hypomethylation seem to precede the somatic genetic alterations in cancer [21,63], the possible use of SST1 methylation as an early detection marker deserves further investigation. The asymmetric association of SST1 demethylation with *TP53* mutations is intriguing. Moderate demethylation may contribute to tumorigenesis in a TP53-independent manner, whereas the association of the age-independent severe demethylation with *TP53* mutations points more to a mechanism other than failure to preserve DNA methylation during replication. Whether “severe” demethylation influences the appearance of *TP53* mutations or is a consequence of the TP53 mutated state is an interesting issue that remains to be studied and characterized.

## 4. Materials and Methods

### 4.1. Tissue Specimens and Samples

Surgically-removed frozen tissues of unselected CRC, gastric, and breast cancers, and paired adjacent non-cancerous tissues were obtained from the Cooperative Human Tissue Network (CHTN, a National Cancer Institute supported resource). None of the patients were treated with chemotherapy prior to surgery. Institutional Review Board approval was obtained to procure and analyze tissues. We selected a random subset of 129 cases for this study, mostly previously analyzed for global DNA methylation changes by MS-AFLP [64]. Microsatellite instability (MSI) was determined in all samples as previously described [21]. Frozen tissues from patients with ovarian carcinoma were provided by the Bellvitge Hospital in Barcelona, Spain. Genomic DNA from freshly frozen tissues was prepared by standard phenol–chloroform extraction and ethanol precipitation. RNA from frozen tissue was extracted either by standard phenol–chloroform extraction or QIAGEN RNeasy RNA extraction kit (Qiagen, Düsseldorf, Germany).

### 4.2. Genome Damage Fraction

Arbitrarily primed PCR (AP-PCR) is a comparative analysis of paired normal and tumor tissue DNA fingerprints used to measure DNA copy number alterations, i.e., gains and losses of chromosomes and chromosomal regions (see Figure 2). The total number of alterations, scored as increases and decreases of band intensities, is used as estimation of the GDF. The GDF values used in this study were previously determined in the laboratory [21]. Genomic DNA (50–100 ng) was subjected to AP-PCR amplification in 25 μL of reaction mix: 1 unit of Taq DNA polymerase (Takara, Madison, WI, USA), 10 mM Tris HCl (pH 8.3), 50 mM KCl, 5 mM MgCl_2_, and 1 mM primer. The reaction was performed using primers BLUE and MCG1 and proceeded to the first five-cycle incubation under conditions of low stringency (94 °C for 30 s, 50 °C for 1 min, and 72 °C for 1.5 min), followed by a 25-cycle incubation under conditions of high stringency (94 °C for 15 s, 60 °C for 15 s, and 72 °C for 1 min), and finally an extension step (72 °C for 7 min). To separate the PCR products, the samples were electrophoresed in 5.5% polyacrylamide gels containing 7 M urea at 55 W for 5–6 h. After electrophoresis, the gels were dried and exposed to X-ray films overnight to visualize the fingerprint bands. Primer sequences and annealing temperatures are shown in Table S3.

### 4.3. Cancer Cell Lines and 5-aza-2′-Deoxycytidine Treatment

Cancer cell lines (Caco-2, LS174T, OV90, A2780) were obtained from the American Type Culture Collection (ATCC, Manassas, VA, USA) and maintained under recommended culture conditions. Other ovarian cancer cell lines were obtained from European Collection of Cell Cultures (ECACC). Cultures were supplemented with fetal bovine serum (FBS) (Hyclone, Thermo Fisher Scientific, Waltham, MA, USA) and 1× antimycotic antibiotics (Omega Scientific, Inc., Tarzana, CA, USA). 5-aza-2′-deoxycytidine (5-aza-dC, Sigma-Aldrich, San Louis, MO, USA)) treatment at 2.5 μM was performed for four days and media changed every 24 h with fresh 5-aza-dC.

### 4.4. Molecular Analyses

MS-AFLP, bisulfite sequencing, RT-PCR, and Western blots were performed as previously published [64]. In brief, for MS-AFLP, two pairs of oligonucleotides were annealed overnight at 37 °C to generate NotI (5′–CTCGTAGACTGCGTAGG–3′ and 5′–GGCCCCTACGCAGTCTAC–3′) and MseI (5′–GACGATGAGTCCTGAG–3′ and 5′–TACTCAGGACTCAT–3′) specific adaptors. Digested DNA was ligated to NotI and MseI adaptors with T4 DNA ligase (Roche, Basel, Switzerland) overnight at 16 °C. A primer complementary to the NotI adaptor (NotI primer, see Table S2) was labeled at the 5′ end using ^32^P-γ-ATP (NEN) and T4 polynucleotide kinase (Promega, Madison, WI, USA). Adaptor-ligated template DNA was PCR amplified using ^32^P-labeled NotI primer and one of non-labeled MseI primers (Table S2). PCR samples were heat-denatured and immediately electrophoresed in denaturing gels (Sequagel-6, National Diagnostics, Atlanta, GA, USA). Gels were dried and exposed to X-ray films.

For bisulfite genomic sequencing, 1 μg of genomic DNA was treated with sodium bisulfite using EZ DNA Methylation Kit (Zymo Research, Irvine, CA, USA). PCR was performed following manufacturer indications. Primer sequences and annealing temperatures are shown in Table S2. PCR products were electrophoresed in 2% agarose gels and purified using Qiaquick Gel Extraction Kit (Qiagen) and, thereafter, cloned into pCR^®^2.1-TOPO^®^ (TOPO-TA Cloning Kit, Invitrogen, Carlsbad, CA, USA), transformed into *Escherichia coli* DH10B. Individual positive clones were amplified, and plasmids containing correct-size inserts were subsequently sequenced using M13 reverse primer.

For RT-PCR, total cellular RNA was extracted using TRIzol Reagent (Invitrogen). Retrotranscription with Superscript II enzyme was followed (Invitrogen) and cDNA was amplified using *HELLS* specific primers (for oligonucleotide sequences and annealing temperatures see Table S3).

For Western blots, nuclear and cytoplasmic extracts were obtained by lysing the cell membrane with hypotonic buffer plus fresh protease inhibitors (Complete, Roche) and phosphatase inhibitors (PhosSTOP, Roche). Pelleted nuclei were then lysed with hypertonic buffer (plus inhibitors as before). Protein extracts were resolved in sodium dodecyl sulfate (SDS)-polyacrylamide gels and transferred to PVDF membranes with the iBlot system (Invitrogen). HELLS antibody (sc-28202, Santa Cruz Biotechnology, Santa Cruz, CA, USA) diluted 1/1000 and β-tubulin antibody (sc-9104, Santa Cruz) were incubated after blocking with 5% non-fat milk for 1 h at room temperature. A secondary anti-rabbit antibody conjugated to horseradish peroxidase was used 1: 4000 (DAKO, Agilent technologies, Santa Clara, CA, USA) and membranes were incubated with enhanced chemiluminescence (ECL) system (Amersham, GE lifesciences, Chicago, IL, USA). Films were exposed and developed at different time points.

### 4.5. Chromatin Immunoprecipitation

Nuclei were isolated by hypotonic lysis. Sonication to obtain chromatin fragments of around 500 bp was performed using a Covaris S2 sonicator, (Woburn, MA, USA). Each sample with an equal final concentration of 100 μg chromatin was incubated overnight with 3 μg of H3K9me3 (17–625), H3K27me3 (07–449), antibodies from Millipore (Billerica, MA, USA); HELLS (AB3851) and control IgG (AB46540) from Abcam, Cambridge, UK. DNA-antibody complexes were recovered by adding protein A/G magnetic beads (Millipore). After extensive washing, bound DNA fragments were eluted (1% *w/v* SDS, 0.1 M Na-HCO_3_) and analyzed by quantitative PCR using SYBR Green Master Mix (Applied Biosystems, Thermo Fisher Scientific). ChIP in FFPE tissue was performed with ChIP-IT^®^ FFPE Active Motif kit (Carlsbad, CA, USA) following the manufacturer’s instructions. Precipitated DNA was analyzed by quantitative PCR as explained above for classical ChIP.

### 4.6. Immunofluorescence

Cells were fixed with 4% paraformaldehyde for 10 minutes, permeabilized with 0.25% Triton-X and blocked with 4% bovine serum albumin in phosphate-buffered saline 1 h at room temperature. Immunostaining with anti-HELLS antibody (Santa Cruz Biotechnology) was performed overnight at 4 °C followed by immunostaining with anti α-tubulin conjugated with Alexa Fluor 488 (Invitrogen). Alexa 555-conjugated secondary antibody was used (Invitrogen). ProLong gold anti-fade reagent (Invitrogen) was used to counterstain nuclei (DAPI) and mount the slides.

### 4.7. Statistical Analyses

Statistical analyses were performed using GraphPad Prism 5.0 (GraphPad Software, La Jolla, CA, USA). All values were expressed as mean ± standard deviation. Statistical differences between variables were analyzed with Student’s *t* tests (paired or unpaired), Mann-Whitney *U* test, or analysis of variance (ANOVA), as appropriate. All *p* values were calculated from two-sided statistical tests, and *p* < 0.05 was considered statistically significant.

## Supplementary Material

1
