# Supplementary material for: Helicase Lymphoid-Specific Enzyme Contributes to the Maintenance of Methylation of SST1 Pericentromeric Repeats That Are Frequently Demethylated in Colon Cancer and Associate with Genomic Damage
Source: Epigenomes. Author manuscript; Available in PMC 2019 Dec 20. (PMC6924650; doi:10.3390/epigenomes1010002)
Supplement: 1 [file NIHMS1062431-supplement-1.docx]

Supplementary Materials: Helicase
Lymphoid-Specific Enzyme Contributes to the Maintenance of Methylation of SST1 Pericentromeric Repeats That Are Frequently Demethylated in Colon Cancer and Associate with Genomic Damage

Johanna K. Samuelsson, Gabrijela Dumbovic, Cristian Polo, Cristina Moreta, Andreu Alibés, Tatiana Ruiz-Larroya, Pepita Giménez-Bonafé, Sergio Alonso, Sonia-V. Forcales and Manuel Perucho


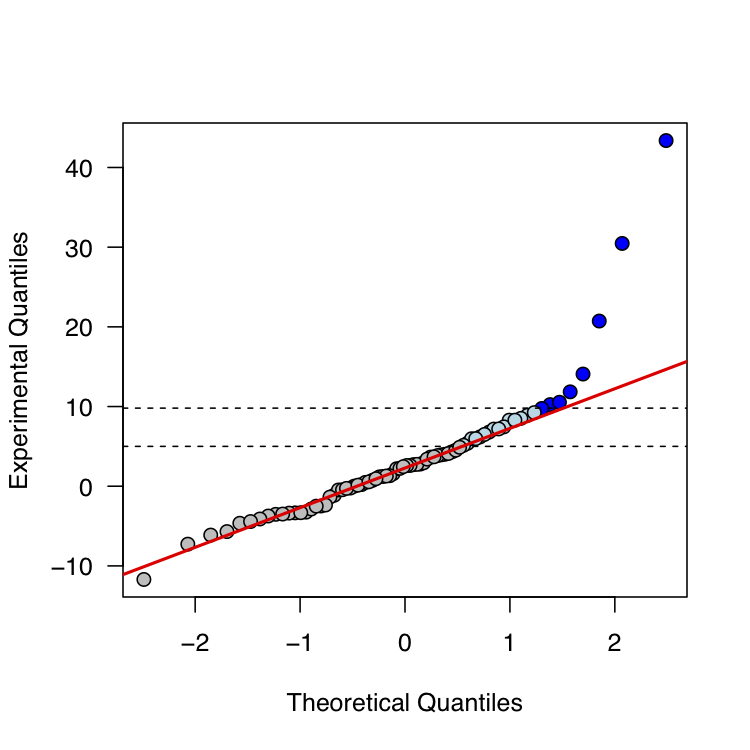


**Figure S1.** Distribution of SST1 somatic demethylation in colorectal cancer (CRC) compared to a theoretical normal distribution. Quantile-quantile plot of normal distribution (*x* axis) vs. the distribution of the SST1 repeat somatic demethylation (difference in mean methylation between normal and matching tumor tissue) estimated by bisulfite sequencing (*y* axis). Dashed lines indicate the cut-off values chosen for classification of tumors with moderate (5%) and severe (10%) demethylation (light and dark blue, respectively). In red, the estimated linear regression line. Graphic performed with R-Studio [1].

| 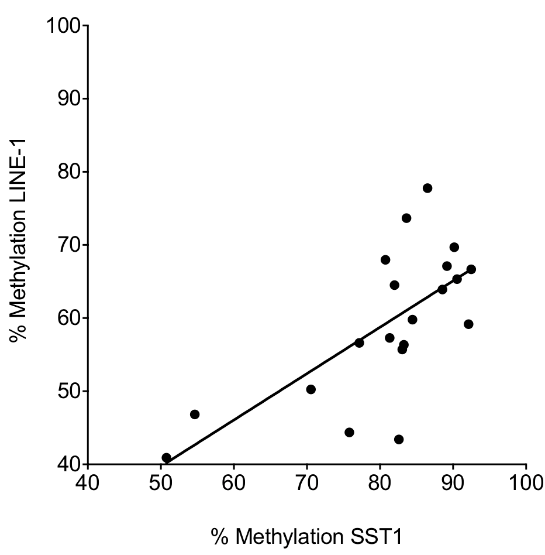 |
| --- |
| (**A**) |
| 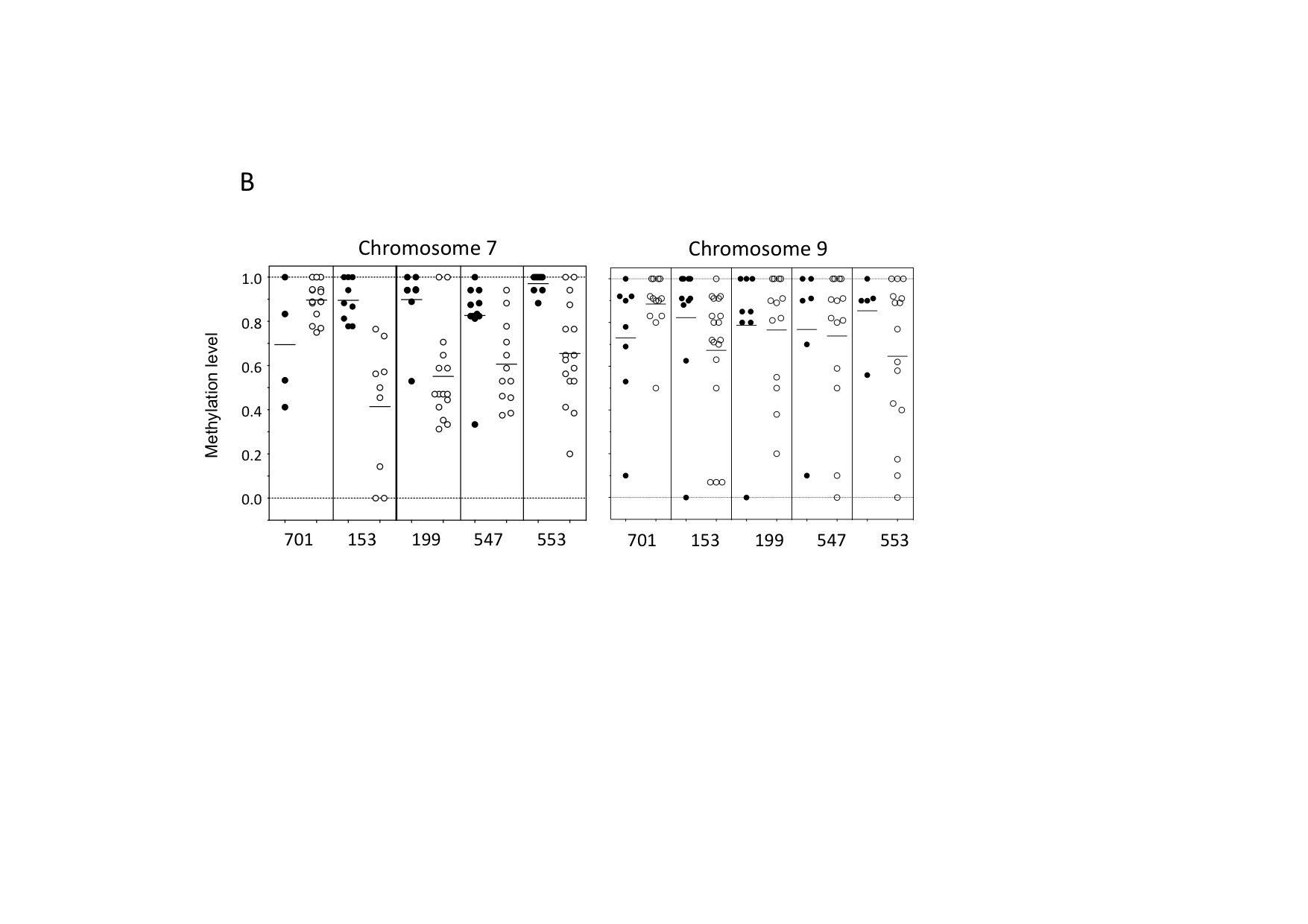 |
| (**B**) |

**Figure S2.** SST1 demethylation is genome-wide. (**A**) SST1 demethylation significantly associates with long interspersed nuclear element-1 (LINE-1) demethylation. Twenty of the colon cancer cases analyzed for SST1 methylation were also analyzed for LINE-1 methylation; and (**B**) methylation status of SST1 satellites from chromosome 7 in CRCs with SST1 demethylation at chromosome 21 (see cases 153, 199, 547 and 553 in Figure 1). Case 701 showed no demethylation of SST1 at chromosome 21. SST1 methylation levels correspond to methylation average of the 28 GCs analyzed by bisulfite sequencing from individual PCR clones in colon normal (black) and tumor (white) samples.


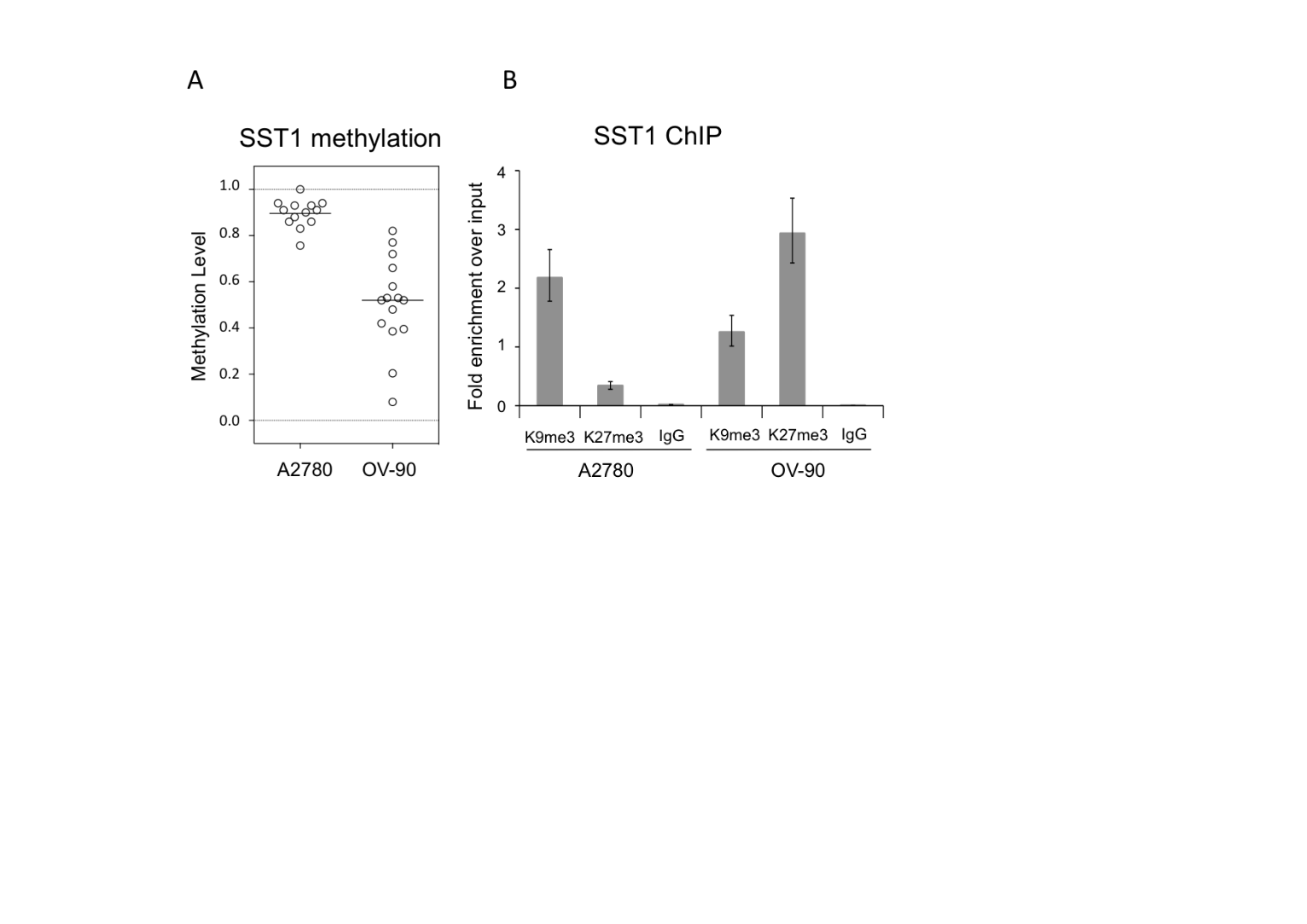


**Figure S3.** Demethylation of SST1 repetitive elements also leads to histone 3 lysine 27 trimethylation (H3K27me3) enrichment in ovarian cancer. (**A**) SST1 methylation levels of individual PCR clones determined by bisulfite sequencing in the ovarian cancer cell lines A2780 and OV-90; and (**B**) chromatin immunoprecipitation (ChIP) analysis of histone modifications associated with highly methylated SST1 in A2780 and SST1 demethylation in OV90. As shown for CRC cell lines, high levels of SST1 methylation (A2780) associate with H3K9me3 and low H3K27me3, while SST1 demethylation associates with decreased H3K9me3 and increased H3K27me3. Fold change is calculated relative to the input. IgG shows immunoprecipitation background levels.

| 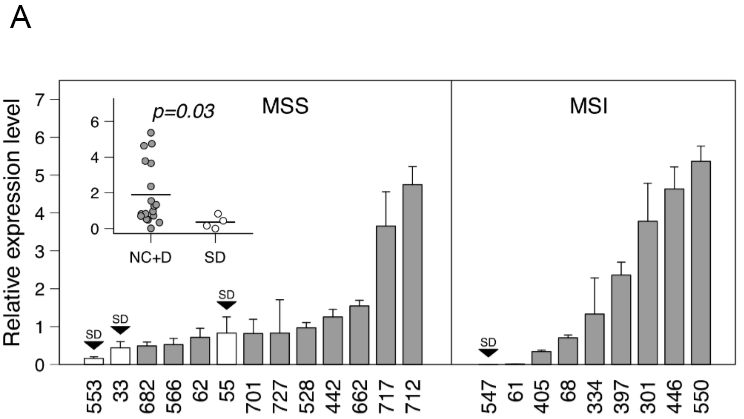 |
| --- |
| **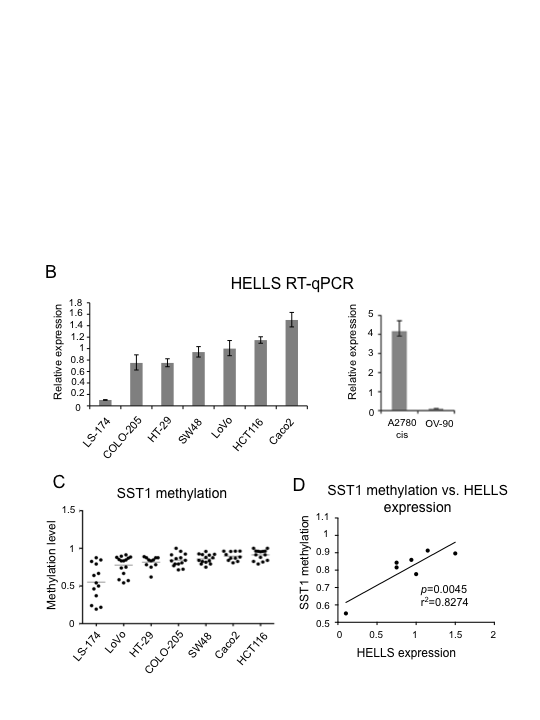** |

**Figure S4.** Helicase lymphoid-specific (HELLS) expression in CRC tumors and cell lines. (**A**) RT-PCR analysis of *HELLS* expression in CRCs with (MSI) and without (MSS) microsatellite instability. Expression values are relative to the expression of B2 microglobulin (×100). Primary tumors exhibiting severe SST1 demethylation are indicated with black triangles (see Figure 1). They consistently displayed low HELLS expression levels. Inserted graph: HELLS expression of colon primary tumors without (NC+D) and with (SD) severe SST1 demethylation (black and white circles, respectively). Performed with R-Studio [1]; (**B**) RT-PCR analysis of HELLS expression in colorectal (left) and ovarian (right) cancer cell lines. The expression was normalized against PUM1 housekeeping gene in CRC cell lines and GAPDH in ovarian cancer cell lines; (**C**) SST1 methylation levels in CRC cell lines analyzed by bisulfite sequencing; and (**D**) correlation between SST1 methylation and HELLS expression in CRC cell lines (from panels B and C).

**Table S1.** SST1 methylation analysis in primary tumors and cancer cell lines.

| **Cancers** | **Number** | **Average SST1 Methylation** | | **SST1 Demethylation** | |
| --- | --- | --- | --- | --- | --- |
|  |  | **Normal Tissue** | **Tumor Tissue** | **Total ^a^** | **Severe ^b^** |
| Colorectal | 128 | 88.7% ± 9.0% | 83.9% ± 15.1% | 22% | 7% |
| Gastric | 88 | ND | ND | 15% | ND |
| Breast | 27 | 83.4% ± 9.4% | 82.5% ± 15.8% | 20% | 15% |
| Ovarian | 25 | 83.3% ± 8.6% | 80.8% ± 19.1% | 20% | 16% |
| **Cell Lines** |  |  |  |  |  |
| Colorectal | 14 |  |  | 64% | 43% |
| Ovary | 9 |  |  | 33% | 33% |

Average methylation of bisulfite sequencing analysis in primary tumors and matched normal tissue from the cancers indicated. Cell lines from colon and ovarian cancers were also analyzed. ND: not determined. Somatic demethylation: difference of DNA methylation levels between normal and tumor tissue over 5% (^a^) and equal or over 10% (^b^).

**Table S2.** SST1 demethylation and CRC clinicopathological features.

|  | **Somatic Demethylation Degree** | | | ***p* Values** | |
| --- | --- | --- | --- | --- | --- |
|  | **No Change** | **Moderate** | **Severe** | ***P1*: NC Vs. Rest** | ***P3*: Sev. Vs. Rest** |
|  | **(<5%)** | **(≥5% <10%)** | **(≥10%)** | ***P2*: NC Vs. Mod.** | ***P4*: Sev. Vs. Mod.** |
| **Age** |  |  |  |  |  |
| <65 | 50 | 6 | 6 | *P1 = 0.22* | *P3 = 0.51* |
| ≥65 | 50 | 13 | 3 | *P2 = 0.12* | *P4 = 0.12* |
| Mean ± SD | 64.2 ± 12.4 | 69.1 ± 12.2 | 63.3 ± 6.6 |  |  |
| **Gender** |  |  |  |  |  |
| Male | 58 | 8 | 8 | *P1 = 1* | *P3 = 0.078* |
| Female | 42 | 11 | 1 | *P2 = 0.22* | ***P4 = 0.039*** |
| **Race** |  |  |  |  |  |
| Afric. Amer. | 29 | 5 | 2 | *P1 = 1* | *P3 = 1* |
| Caucasian | 59 | 11 | 5 | *P2 = 1* | *P4 = 1* |
| **Location** |  |  |  |  |  |
| Proximal | 55 | 13 | 2 | *P1 = 0.83* | *P3 = 0.069* |
| Distal | 39 | 6 | 6 | *P2 = 0.46* | *P4 = 0.088* |
| **Stage** |  |  |  |  |  |
| A+B | 53 | 10 | 6 | *P1 = 0.83* | *P3 = 0.51* |
| C+D+M | 46 | 9 | 3 | *P2 = 1* | *P4 = 0.69* |
| **Survival** |  |  |  |  |  |
| Alive | 24 | 3 | 6 | *P1 = 0.84* | *P3 = 0.18* |
| Dead | 27 | 8 | 2 | *P2 = 0.46* | *P4 = 0.08* |
| Median months | 48.8 | 30.3 | NA |  |  |
| **MSI** |  |  |  |  |  |
| Negative | 80 | 15 | 8 | *P1 = 1* | *P3 = 1* |
| Positive | 20 | 4 | 1 | *P2 = 1* | *P4 = 1* |
| ***KRAS*** |  |  |  |  |  |
| Negative | 60 | 11 | 8 | *P1 = 0.82* | *P3 = 0.16* |
| Positive | 34 | 8 | 1 | *P2 = 0.61* | *P4 = 0.20* |
| ***TP53*** |  |  |  |  |  |
| Negative | 55 | 12 | 1 | *P1 = 0.40* | ***P3 = 0.012*** |
| Positive | 43 | 7 | 8 | *P2 = 0.62* | ***P4 = 0.016*** |

*p* Values were calculated by Fisher’s exact test except for age and survival, where Student’s *t* test and log-rank test were applied, respectively. *p* Values were rounded to a maximum of two significant digits. In bold type, *p* values < 0.05. For every clinical or molecular variable, four comparisons were performed according to the tumor’s methylation status (i.e., *P1*: no change in methylation (NC) vs. the rest: moderate (Mod.) plus severe (Sev.) demethylation). NC: no change in methylation (<5%). Mod: moderate demethylation (5%–10%). Sev: severe demethylation (≥10%). NA: not applicable. In a logistic regression multivariate analysis of severe demethylation tumors (*n* = 9) vs. the rest (*n* = 119), including age, gender, and *TP53* as explanatory factors, *TP53* mutations retained statistical significance (*p* = 0.034), with a borderline significant value (*p* = 0.1) for the association with the male gender. When using the 10% cutoff, the difference in age was not significant. The severely-demethylated tumors associated with younger aged patients, but only when a slightly higher cutoff threshold was employed to classify tumors into the severe demethylated category (Figure 3).

**Table S3.** Primer sequences, annealing temperatures, and amplicon size.

| **Methylation** | **Forward (5′ to 3′)** | **Reverse (5′ to 3′)** | **T_m_ (°C)** | **Size** | **Method** |
| --- | --- | --- | --- | --- | --- |
| NotI | GACTGCGTAGGGGCCGCG |  | 52 | N/A | MS-AFLP |
| MseI-C |  | GATGAGTCCTGAGTAAC | 52 | N/A | MS-AFLP |
| MseI-CA |  | GATGAGTCCTGAGTAACA | 52 | N/A | MS-AFLP |
| MseI-CG |  | GATGAGTCCTGAGTAACG | 52 | N/A | MS-AFLP |
| SST1 | GGGGATTGGTGTTTAAGATA | AAAACTCCCCCTCCCTTAATAA | 55 | 317 | Bis.Seq |
| SST1-7 | GGGGATTGGTGTTTAAGATA | TCCTAAAAATTTAACCCTTTAAC | 56 | 257 | Bis.Seq |
| SST1-9 | GGGGATTGGTGTTTAAGATA | AAACCTAAAATCCCATTTCCT | 56 | 268 | Bis.Seq |
| LINE-1 | ACAAACAAACCTCCTTAAACT | ACAAACAAACCTCCTTAAACT | 55 | 418 | Bis.Seq |
| SATa | GTATATGTAAGTGGATATTGG | ATTAAACTCAATCATCCC | 55 | 282 | Bis.Seq |
| **GDF** |  |  |  |  |  |
| MCG1 | AACCCTCACCCTAACCCCAA |  | 50–60 | N/A | AP-PCR |
| BLUE | CCGAATTCGCAAAGCTCTGA |  | 50–60 | N/A | AP-PCR |
| **ChIP** |  |  |  |  |  |
| SST1 | AACCACTGTGACGGGAGAAA | CTGGGACAGGACGAGACAC | 55 | 219 | ChIP, FFPE ChIP |
| SATα | AAGGTCAATGGCAGAAAAGGA | CAACGAAGGCCACAAGATGTC | 55 | 183 | ChIP |
| GAPDH | TCGACAGTCAGCCGCATCT | CTAGCCTCCCGGGTTTCTCT | 55 | 69 | FFPE ChIP |
| ALU | GGATCACCTGAGGTCAGGAGT | CCCAGGCTGGAGTGCAGTGGC | 55 | 203 | ChIP |
| **Expression** |  |  |  |  |  |
| HELLS | GCTTGATGGGTCCATGTCTT | GACATCTATCCTGGGCCTGA | 60 | 194 | qPCR |
| PUM1 | CGGTCGTCCTGAGGATAAAA | CGTACGTGAGGCGTGAGTAA | 55 | 121 | qPCR |
| B2M | CCAGCAGAGAATGGAAAGTC | GATGCTGCTTACATGTCTCG | 60 | 269 | qPCR |
| GAPDH | GAAGGTGAAGGTCGGAGT | GAAGATGGTGATGGGATTTC | 55 | 226 | qPCR |

GDF: genomic damage fraction; MS-AFLP: methylation sensitive-amplified fragment length polymorphism; Bis.Seq: bisulfite sequencing; AP-PCR: arbitrarily-primed PCR; ChIP: chromatin immunoprecipitation; FFPE: formalin-fixed paraffin embedded; qPCR: quantitative PCR; N/A: not applicable.

Reference

1. R Core Team. *A Language and Environment for Statistical Computing*; R Foundation for Statistical Computing: Vienna, Austria, 2015.
